# Cilostazol protective effect on nedaplatin-induced genotoxicity in cultured human lymphocytes

**DOI:** 10.1016/j.toxrep.2025.101928

**Published:** 2025-01-25

**Authors:** Karem H. Alzoubi, Abeer M. Rababa’h, Omar F. Khabour, Fian Nuseir

**Affiliations:** a Department of Pharmacy Practice and Pharmacotherapeutics, College of Pharmacy, The University of Sharjah, Sharjah 27272, United Arab Emirates; b Department of Clinical Pharmacy, Faculty of Pharmacy, Jordan University of Science and Technology, Irbid, Jordan; c Division of Molecular Pharmacology and Experimental Therapeutics, Mayo Clinic, 200 First St SW, Rochester, MN 55902, USA; d Department of Medical Laboratory Sciences, Faculty of Applied Medical Sciences, Jordan University of Science and Technology, Irbid, Jordan

**Keywords:** Nedaplatin, Cilostazol, Mitotic index, Human cultured lymphocytes, Sister chromatid exchanges, Genotoxicity

## Abstract

**Background:**

Nedaplatin has demonstrated remarkable efficacy in combating various malignancies. However, the administration of nedaplatin has been associated with the induction of DNA damage within normal cells. Cilostazol is a phosphodiesterase III inhibitor with an antioxidant mechanism that could protect cells from genotoxicity. We aimed to evaluate the genotoxic effect of nedaplatin on cultured human lymphocytes and the potential protective effect of cilostazol on chromosomal damage induced by nedaplatin.

**Methods:**

The proposed nedaplatin’s genotoxic effect was studied *in vitro* by evaluating the frequencies of sister chromatid exchanges (SCEs) in human cultured lymphocytes. Both the mitotic and proliferative indices (MI and PI, respectively) were used to assess the cytotoxic effects of nedaplatin.

**Results:**

Nedaplatin significantly increased the frequency of SCEs compared to control and cilostazol-treated cells. The chromosomal injury induced by nedaplatin was significantly reduced by pretreatment of cells with cilostazol (P < 0.0001). Treating with cilostazol alone also reduced the frequency of SCEs, MI, and PI compared to the control group. Nedaplatin induced significant decreases in the MI and PI compared to the control group. Pretreatment with cilostazol partially debilitated the nedaplatin-induced changes in MI but not PI.

**Conclusion:**

Cilostazol ameliorated the genotoxicity of nedaplatin in cultured human lymphocytes.

## Highlights

• Nedaplatin induces DNA damage within normal cells.
• Cilostazol is a phosphodiesterase III inhibitor that could protect cells from genotoxicity.
• The potential protective effect of cilostazol on chromosomal damage induced by nedaplatin was studied.
• Cilostazol ameliorated the genotoxicity of nedaplatin in cultured human lymphocytes.

## 1 Introduction

Cancer treatment includes different approaches, such as surgery, radiation, bone marrow transplantation, and chemotherapy. In the chemotherapy approach, one or more anti-cancer agents are usually engaged in the therapeutic protocols. Nedaplatin, a second-generation platinum-containing chemotherapeutic agent extensively employed in cancer therapy, has demonstrated remarkable efficacy in combating various solid malignancies [4], [6]. The therapeutic efficacy of nedaplatin lies in its ability to inhibit cancer cell growth and division by forming covalent bonds with DNA, leading to the formation of cross-links within and between DNA strands [25], [30]. These DNA cross-links interfere with DNA replication and transcription processes, ultimately inducing cell cycle arrest and apoptosis in cancer cells [20], [24], [4], [6]. Platinum-based anticancer agents also possess genotoxic properties that can increase the risk of developing secondary cancers in normal cells via mechanisms involving induction of oxidative stress [14]. For example, platinum agents has been shown to induce oxidative stress in normal human lymphocytes, plasma, and blood platelets [7]. In the case of nedaplatin, recent studies have revealed that its genotoxic effect could be primarily mediated by initiating oxidative stress pathways [3], [8], [9]. Therefore, there is a critical need to explore adjunctive therapeutic interventions that can ameliorate the genotoxicity caused by nedaplatin.

Cilostazol, a selective phosphodiesterase III inhibitor, is primarily used as an antiplatelet agent to manage peripheral vascular diseases [42]. Beyond its cardiovascular benefits, cilostazol has been shown to possess diverse pharmacological properties, including anti-inflammatory, antioxidant, and cytoprotective effects [35]. Cilostazol has been shown to trap reactive oxygen species and inhibit the cell damage induced by oxidative stress, in addition to inhibiting lipid peroxidation in a model of cerebral ischemia [23], [33]. We showed previously that cilostazol lowered the chromosomal damage induced by methotrexate and reduced the mitotic index significantly due to its anti-proliferative effect [34]. Hence, emerging evidence suggests that cilostazol and other antioxidant agents may potentially reduce genotoxicity induced by various genotoxic agents [19], [21], [28], [34], [36].

In the current study, the genotoxicity of nedaplatin was investigated using sister chromatid exchange (SCE) assay in cultured human lymphocytes. Moreover, the effect of cilostazol in ameliorating nedaplatin genotoxicity was also assessed. Preventing the genotoxic effects of anticancer drugs on normal cells can effectively mitigate the risk of chemotherapy-induced secondary cancers.

## 2 Materials and methods

### 2.1 Subject

In the current study, peripheral venous blood was collected in sodium heparin tubes from male subjects (n = 5; age range 20–30 years old). The participants in the study refrained from using drugs or alcohol, abstained from smoking cigarettes or waterpipe tobacco, and were not taking any medications. The blood samples were obtained from donors on the same day of the experiments, before any dietary intake, to minimize potential dietary effects. All participants were provided with a detailed explanation of the study's aims and objectives, and their written informed consent was obtained before participating. The study received approval from the Institutional Review Board of Jordan University of Science and Technology (IRB approval no. 27/132/2020; Dated: 31/3/2020). Furthermore, the present study was conducted by the guidelines outlined in the Declaration of Helsinki for research involving human subjects [41].

### 2.2 Reagents

The PB-MAX karyotyping medium and Gurr buffer were procured from Gibco Invitrogen (Paisley, UK). Colcemid was obtained from EuroClone (S.P.A) (Siziano, Italy). Nedaplatin (CAS number: 95734–82–0, purity >98 %; Sigma-Aldrich (St. Louis, MO)), Cilostazol (CAS number:73963–72, purity >98 %; Santa Cruz Biotechnology, USA)), and other reagents were purchased from Sigma-Aldrich (St. Louis, MO). The materials were sourced through local agents in the Middle East.

### 2.3 Human lymphocyte cultures

Cultures of human lymphocytes were initiated by adding 1 mL of whole blood into 9 mL PB-MAX medium in sterile 50 mL culture flasks. The PB-Max is an optimized, fully supplemented RPMI 1640 medium that comprises 15 % fetal bovine serum, L-glutamine, and phytohaemagglutinin [34]. Cultures were used for cytogenetic assays as shown below.

### 2.4 Sister-chromatid exchange assay

For the sister-chromatid exchange assay, 5-bromodeoxyuridine at a final concentration of 10 µg/mL was added to cultured lymphocytes directly after culture initiation [3]. The culture flasks were then maintained in a proper incubator at 37°C with 5 % CO_2_ during the experimental period of 72 h. Cultures were distributed into 4 groups: control, nedaplatin (1 µg/mL), cilostazol (1.2 μg/mL), and the combination of nedaplatin and cilostazol (nedaplatin+cilostazol). The concentration of nedaplatin used in this study falls within the range previously demonstrated to induce genotoxicity in cultured human cells [15]. Freshly prepared nedaplatin was dissolved in the culture media to create a stock solution with a concentration of 1 mg/mL. The concentration of cilostazol was based on a previous study that established the protective effect of the used concentration against methotrexate-induced toxicity [34]. Cilostazol prepared in dimethyl sulfoxide was added to lymphocyte cultures directly after culture establishment. Nedaplatin was added to cultures during the last 24 h of 72 h-experimental period [4]. Both the control cultures and the group treated with nedaplatin alone were also treated with an equal amount of the vehicle used in the cilostazol groups.

At the end of the culture period (72 h), lymphocytes were harvested by treating cultures with colcemid at a final concentration of 10 mg/mL [2] for two hours, effectively arresting the cells in the mitotic phase. Cultures were then transferred into 15 mL conical tubes, centrifuged at 500xg for 5 min, and supernatants were discarded. Subsequently, the cultures were exposed to a hypotonic solution (0.075 M) for 30 min. Afterward, cultures were centrifuged at 500xg for 5 min and the supernatants were discarded. The cellular pellets were then fixed and washed three times using a methanol/acetic acid (3/1 by volume) fixative, following the established protocol [21], [29], [37]. The cellular pellets were re-suspended in 1.5 mL of the indicated fixative. Well-spread metaphase chromosomes were obtained by carefully dropping the cellular suspension onto microscopic slides.

The staining of chromosomes to determine the frequency of SCEs was performed using the Hoechst plus Giemsa procedure, following the previously described method [10]. This involved incubating slides containing chromosomal metaphase spreads in Hoechst 32285 dye solution (final concentration 10 µg/mL in distilled water) for 20 min [5]. Slides were then washed with distilled water and then incubated in Gurr buffer under UV light (wavelength 365 nm) for 20 min [12]. Slides were then washed with distilled water and then stained with 5 % Giemsa stain in phosphate buffer (pH 6.8) for 5 min. Approximately 50 well-spread metaphase II (M2) cells per donor were analyzed for the presence of SCEs. These M2 cells contained a range of 42–46 chromosomes. As the study included five donors, 250 cells were scored for SCEs in each treatment group. The scoring was conducted using a high-resolution light microscope (Nikon Corporation) with immersion oil at a magnification of 1000, following the previously mentioned methodology [37].

### 2.5 Cell kinetic indices

To determine the proliferative index (PI), a total of 250 cells in metaphases I, II, and III were used, following the methodology described in a previous study [27]. The PI was calculated using the formula: (1 * MI cells + 2 * MII cells + 3 * MIII cells) / 100 %, where M1, M2, and M3 represent the number of cells at the first, second, and third metaphases, respectively. To compute the mitotic index (MI), at least 1000 cells per treatment per donor were examined. The MI was determined by dividing the percentage of cells in the metaphase stage by the total number of cells [11].

### 2.6 Statistical analysis

Data analysis was achieved using GraphPad Prism version 7 (GraphPad Software, Inc.). Sister-chromated exchange levels, mitotic index, and proliferative index were expressed as means ± standard deviation. Since multiple groups (4 groups) were compared and the data were normally distributed, a one‑way ANOVA followed by Tukey's post hoc analysis was used. P < 0.05 was considered to indicate a statistically significant difference.

## 3 Results

**Fig. 1:**
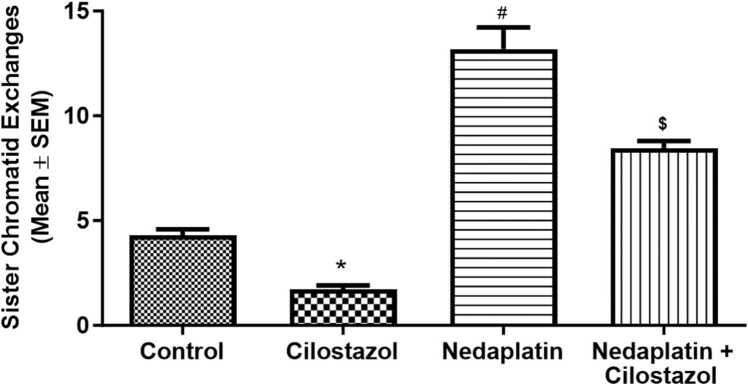
The effects of nedaplatin and cilostazol on the frequency of sister chromatid exchanges (SCEs) in cultured human lymphocytes. Nedaplatin treatment resulted in a significant increase in SCE frequency. Cilostazol alone did not have an impact on SCE frequency. However, when combined with nedaplatin, cilostazol reduced the frequency of SCE induced by nedaplatin. Data are presented as the mean ± SEM. *P < 0.05 vs. control group; #P < 0.05 vs. cilostazol group; $P < 0.05 vs. nedaplatin group.

Fig. 1 illustrates the SCE frequency of the various experimental groups. Treatment with nedaplatin significantly increased the SCE frequency in cultured human lymphocytes (P < 0.0001). The cilostazol-treated group exhibited SCE levels lower than the control group (p < 0.05; Fig. 1). Pre-treating the cultures with cilostazol reduced the frequency of nedaplatin-induced SCE by approximately 35.8 % (p < 0.0001; Table 1). Nevertheless, the frequency of SCE in the combination (nedaplatin and cilostazol) group remained higher than the level observed in the control group (p < 0.05). Therefore, while cilostazol significantly reduced the SCE frequency induced by nedaplatin, it did not completely normalize it.

**Table 1:** Sister chromatid exchanges frequency by different treatment in cultured human lymphocytes.

**Donor/Treatment**	**Frequency of sister Chromatid** **Exchanges**
**Donor1**	
Control Cilostazol Nedaplatin Nedaplatin+ Cilostazol	4.28 2.76 16.5 8.432
**Donor2**	
Control Cilostazol Nedaplatin Nedaplatin+ Cilostazol	4.36 2.14 13.2 8.721
**Donor3**	
Control Cilostazol Nedaplatin Nedaplatin+ Cilostazol	4.18 1.9 10.46 9.435
**Donor4**	
Control Cilostazol Nedaplatin Nedaplatin+ Cilostazol	4.26 1.88 14.14 7.324
**Donor5**	
Control Cilostazol Nedaplatin Nedaplatin+ Cilostazol	4.52 1.94 11.6 8.349
**Total Mean**	
Control Cilostazol Nedaplatin Nedaplatin+ Cilostazol ***ANOVA P-Value***	4.32 ± 0.058 2.124 ± 0.166 13.18 ± 1.045^a^^,^^b^ 8.452 ± 0.341^a^, ^b^ < 0.0001

Data expressed as mean±SEM.

a Significantly different from the control group.

b Significantly different from cilostazol group.

**Fig. 2:**
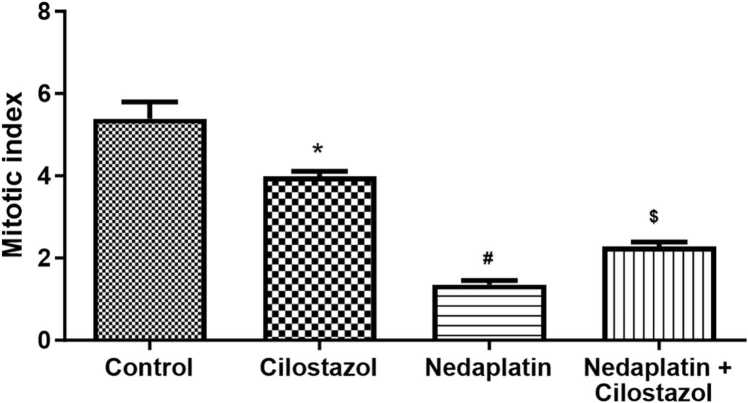
The effects of nedaplatin and cilostazol on the mitotic index (MI) in cultured lymphocytes. Nedaplatin treatment led to a significant reduction in MI. Pretreatment with cilostazol mitigated the decline caused by nedaplatin, although it could not restore it to the control level. Data are presented as the mean ± SEM. *P < 0.05 vs. control group; #P < 0.05 vs. cilostazol group; $P < 0.05 vs. nedaplatin group.

**Fig. 3:**
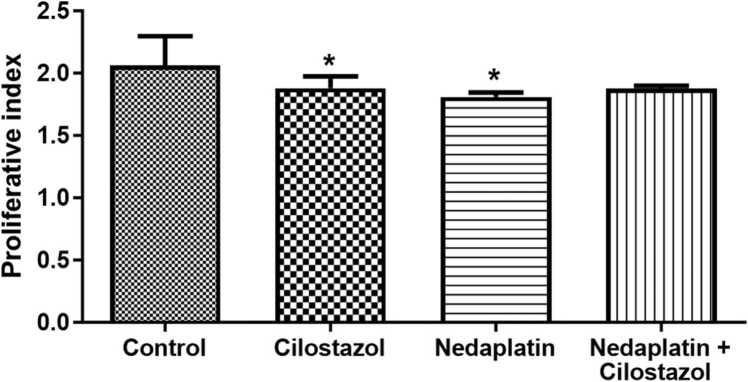
The effects of nedaplatin and cilostazol on the proliferative index (PI). the proliferative indices were slightly reduced in the nedaplatin, cilostazol, and nedaplatin + cilostazol groups. Data are presented as the mean ± SEM.

The impact of cilostazol on the cytotoxicity of nedaplatin was evaluated by examining the mitotic index (MI) and proliferative index (PI) (Fig. 2, Fig. 3, respectively). Treatment with nedaplatin significantly decreased the MI (p < 0.0001; Fig. 2). Pretreating the human lymphocyte cell cultures with cilostazol significantly influenced the reduction in MI caused by nedaplatin (p < 0.0001), although it did not fully restore it to normal levels. Regarding the PI, nedaplatin and/or cilostazol treatments resulted in a significant reduction compared to the control group (p < 0.05; Fig. 3).

## 4 Discussion

The present study aimed to investigate the genotoxic effects of the anticancer drug nedaplatin. This was accomplished by conducting sister chromatid exchange (SCE) on cultured normal human lymphocytes. The findings revealed that nedaplatin exhibited genotoxicity in lymphocyte cells, and pretreating the lymphocytes with cilostazol reduced this genotoxicity.

Previous research has demonstrated that administering highly effective anticancer agents can result in direct cellular toxicity [20], [24], [6]. Additionally, studies have shown that using different anticancer agents in experimental settings can lead to carcinogenic, teratogenic, and mutagenic effects. For instance, nedaplatin has been reported to exhibit genotoxic effects on specific human lung cancer and osteosarcoma cell lines [15]. One of the proposed mechanisms for the genotoxic effects of nedaplatin is its ability to induce DNA cross-linking [26], [38]. Furthermore, nedaplatin has been shown to cause DNA damage by triggering the production of reactive oxygen species (ROS) in nasopharyngeal carcinoma cells [31]. The data obtained from the present study revealed that treatment with nedaplatin (1 μg/mL) resulted in genotoxicity in normal healthy blood cells, as evidenced by a significant increase in the frequency of SCE. This finding is consistent with previous studies that have reported the genotoxic effects of nedaplatin [15]. For example, a previously published report has exhibited a considerable increase in the frequency of SCE in cultured human lymphocytes from healthy individuals exposed to nedaplatin [4].

Cilostazol, a specific reversible inhibitor of PDE III with anti-platelet and vasodilation effects, has been shown to reduce oxidative stress by decreasing the production of ROS [13]. Cilostazol was found to have a protective effect against cadmium-induced cytotoxicity in cultured vascular endothelial cells by increasing cellular metallothionein levels [18]. Metallothionein belongs to the small cysteine-rich protein that is involved in metal homeostasis and is reported to protect cells against cellular toxicity, DNA damage, and oxidative stress [40]. In addition, cilostazol treatment significantly reduced the percentage of 8-OHdG, a marker for oxidative DNA damage, positive neurons in a mouse model of permanent focal ischemia [39]. The current study demonstrated that pretreating lymphocyte cells with cilostazol alleviated the chromosomal damage caused by nedaplatin in cultured human lymphocytes. These results align with a previous study that has shown a similar protective effect of cilostazol against the genotoxicity induced by methotrexate in human cultured lymphocytes [34]. In addition, the current report revealed that cilostazol reduced the frequency of spontaneous SCE compared to the control group. This suggests that cilostazol may have a role in decreasing the spontaneous occurrence of SCE, potentially by suppressing the basal level of oxidative stress and enhancing DNA repair by overexpression metallothionein [17], [40]. The antioxidant potential of cilostazol has been demonstrated in different systems including animal models in a dose dependent manner [16], [22], [32]. For example, cilostazol, in a dose-dependent manner, has been shown to protect against cisplatin-induced testicular toxicity through overcoming oxidative stress and inflammation, and apoptosis [32]. In addition, cilostazol has been shown to exert long-term protection against oxidative stress and inflammatory damage in Type II diabetic patients [1]. However, further investigations are required to elucidate the precise mechanism underlying this reduction effect.

The study’s results indicated that nedaplatin exhibited cytotoxic effects on cultured human lymphocytes, as evidenced by a significant reduction in the mitotic index compared to the control group. *In vitro* studies conducted on human cancer cells have also reported a positive correlation between the cytotoxicity index and the administration of nedaplatin alone or in combination with other chemotherapeutic agents [15]. Furthermore, the results demonstrated that cilostazol reduced the cytotoxicity induced by nedaplatin. These findings suggest that further investigations should be utilized to understand better the mechanisms involved in the modulation of genotoxicity and cytotoxicity by cilostazol.

The current study has limitations, including subjectivity in scoring the genotoxicity/cytotoxicity parameters (sister chromatid exchanges, mitotic index, and proliferative index), which can be avoided using a blind study design in future experiments. Thus, further investigations using more *in vitro* and *in vivo* approaches including preclinical studies are necessary to validate the findings. Additionally, the genotoxicity of nedaplatin was observed 24 h after treatment with a single dose of 1 μg/mL. Therefore, future studies should include dose-response curves and longer durations are needed to provide a more comprehensive understanding. Furthermore, limited research on the genotoxicity of cilostazol itself warrants further studies in this area. These should include exploring the possible mechanisms by which cilostazol exerts its genotoxic protective effects. Considering the protective effects of cilostazol observed in this study, potential clinical applications or further preclinical studies should investigate cilostazol for mitigation of some of the adverse effects of nedaplatin chemotherapy.

To summarize, the present study has contributed to our understanding of the genotoxic effects of nedaplatin and provided supportive evidence of its ability to induce genotoxicity in normal human cells. Importantly, pretreatment of lymphocytes with cilostazol offered protection against the genotoxicity induced by nedaplatin. This suggests that cilostazol could be utilized to mitigate some of the adverse effects of nedaplatin on normal cells.

## Ethics and consent declarations

The study received approval from the Institutional Review Board of Jordan University of Science and Technology (IRB approval no. 27/132/2020; dated 31/3/2020). Furthermore, the present study was conducted according to the guidelines outlined in the Declaration of Helsinki for research involving human subjects. No animals were used for studies that are the basis of this research.

## Funding

This research was funded by the Deanship of Research at 10.13039/501100004035Jordan University of Science and Technology, Irbid, Jordan (Grant no. 252/2020).

## CRediT authorship contribution statement

**Khabour Omar F.:** Writing – review & editing, Writing – original draft, Visualization, Supervision, Resources, Project administration, Methodology, Investigation, Formal analysis, Data curation, Conceptualization. **Nuseir Fian:** Writing – original draft, Visualization, Validation, Project administration, Methodology, Investigation, Data curation, Conceptualization. **Alzoubi Karem:** Writing – review & editing, Writing – original draft, Validation, Supervision, Resources, Project administration, Methodology, Investigation, Funding acquisition, Data curation, Conceptualization. **Rababa’h Abeer M.:** Writing – review & editing, Writing – original draft, Visualization, Validation, Supervision, Resources, Project administration, Methodology, Investigation, Funding acquisition, Formal analysis, Data curation, Conceptualization.

## Declaration of Competing Interest

The authors declare that they have no known competing financial interests or personal relationships that could have appeared to influence the work reported in this paper.

## Data Availability

Data will be made available on request.
